# Supplementary material for: Trust in government moderates the association between fear of COVID-19 as well as empathic concern and preventive behaviour
Source: Commun Psychol. 2023 Dec 15;1:43. doi: 10.1038/s44271-023-00046-5 (PMC11332001; doi:10.1038/s44271-023-00046-5)
Supplement: Supplementary file 1 — Supplementary Information [file 44271_2023_46_MOESM1_ESM.pdf]

Supplementary Information

Supplementary Note 1

The comparison of the results obtained under the three different methodological approaches revealed that results from the OLS regression approach (Model 1) confirmed the results that we obtained when using a random-slopes MLM approach (Model 1). In both types of regression models, the interaction between trust in the government on the country-level and fear of COVID-19 was found significant, while the statistical analyses did not provide evidence for a significant interaction between country-level trust in the government and empathic prosocial concern (see Supplementary Table 2). However, when applying the cluster-robust standard errors correction to the OLS regression (Model 3), the results obtained from this analysis did no longer support the existence of a significant interaction between trust in the government and fear of COVID-19. Hence, while Hypothesis 2b seems supported under Model 1 and Model 2, the analysis results obtained under Model 3, where the size of standard errors is typically larger, do not support this hypothesis.

While this difference in results between Model 1 and Model 3 may be interpreted as an indicator for a less robust interaction effect, it should also be noted that there is an ongoing discussion concerning the use of these two regression approaches (MLM vs. OLS with cluster-robust errors). Whether one or the other approach should be preferred depends much on the research setting and the type of research question that is to be tested. While the OLS with cluster-robust standard errors seems more appropriate for single-level models where the researcher theorizes variation across different units (i.e., countries) more like a nuisance factor that must be controlled for, the MLM approach seems more appropriate in settings that assume a multi-level structure where the different units are more independent from each other and can produce different effects (for a critical comparison, see[1]). We thus believe that the random-effects MLM approach (Model 1) that we reported in the main analyses is more

suitable for testing the hypotheses in the current research setting. Regression estimates under the different regression models are presented in Supplementary Table 2.

Supplementary Table 1. Comparison of results under different regression models.

	Model 1: Initial model (Multi-level Modelling; MLM)	Model 2: Basic OLS regression	Model 3: OLS regression with cluster-robust standard errors
	B(SE)	B(SE)	B (SE)
Intercept	3.75 (0.09) ***	3.71 (0.01) ***	3.71 (0.08)***
TG (Country-Level)	0.01 (0.004)	0.01 (0.0001) ***	0.01 (0.004)*
EC	0.13 (0.01) ***	0.14 (0.01) ***	0.14 (0.01)***
FoC	0.31 (0.03) ***	0.30 (0.01) ***	0.30 (0.03)***
TG (Country-Lev.) × EC	-0.0003 (0.0003)	-0.0001 (0.0001)	0.0001 (0.0001)
TG (Country-Lev.) × FoC	-0.002 (0.001) **	-0.001 (0.0001) ***	-0.001 (0.001)
EC × FoC	-0.04 (0.01) ***	-0.03 (0.01) ***	-0.03 (0.01)**

Notes. N = 11,026 independent responses nested in 29 countries; TG = Trust in Government, EC = Empathic Concern, FoC = Fear of COVID-19. ** $p < 0.01$, *** $p < 0.001$. All analyses were performed by entering covariate effects of gender, age, HDI (Human Development Index), hospital beds per 1000, month of data collection, government stringency level, and the number of new daily COVID-19 cases and deaths by the time of data collection. For reasons of simplicity, the covariate effects are not displayed in the table.

Supplementary Note 2

Below we present relevant descriptive statistics for the dependent variable support for COVID-19 containment behaviors, the data distribution of the present sample as histogram, and the Q-Q plot for the dependent variable support for COVID-19 containment behaviors based on the total sample consisting of N = 12,758 responses. Due to the large sample size of the present study, the normality of the data distribution was not formally tested.

Supplementary Table 2. Descriptive statistics for supporting COVID-19 containment behaviors.

	Support for COVID-19 containment behaviors
Mean	3.712
SD	0.925
Skewness	-0.742
SE of Skewness	0.022
Kurtosis	-0.021
SD of Kurtosis	0.043
Minimum	1.000
Maximum	5.000

Notes. Statistics refer to $N = 12{,}758$ independent responses; SD = Standard Deviation; SE = Standard Error.

Supplementary Figure 1. Histogram for supporting COVID-19 containment behaviors.

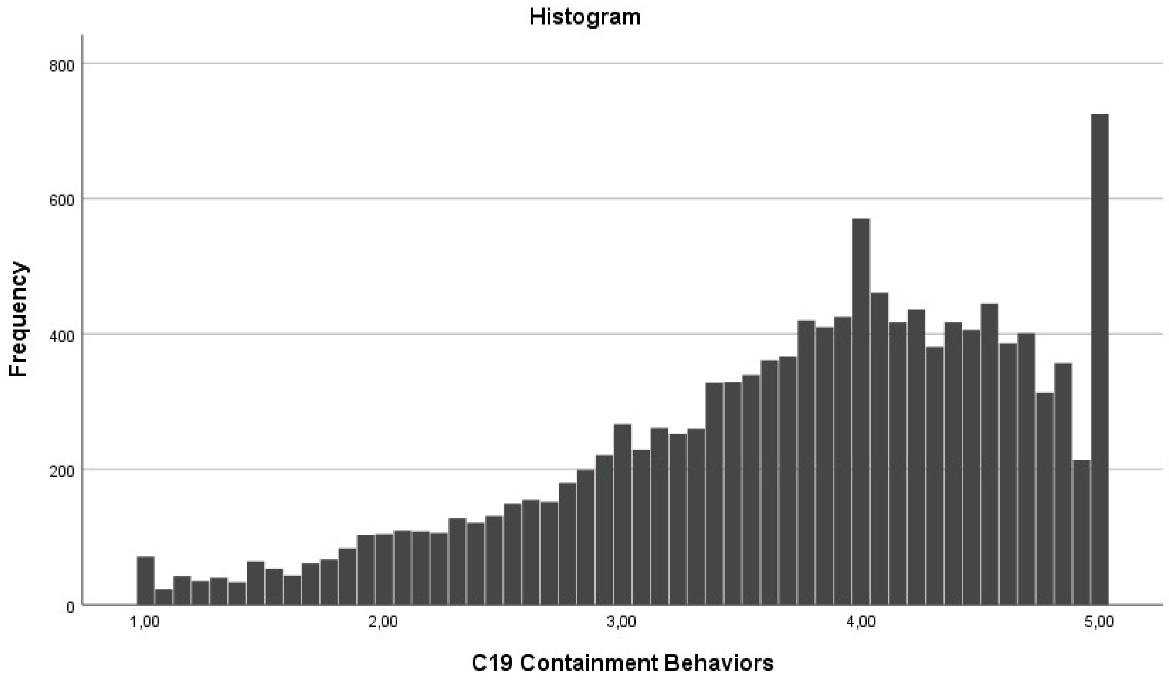

Notes. Histogram displays the frequencies for the mean scores obtained for self-reported support for COVID-19 containment behaviors across $N = 12{,}758$ independent respondents.

Supplementary Figure 2. Q-Q plot for supporting COVID-19 containment behaviors.

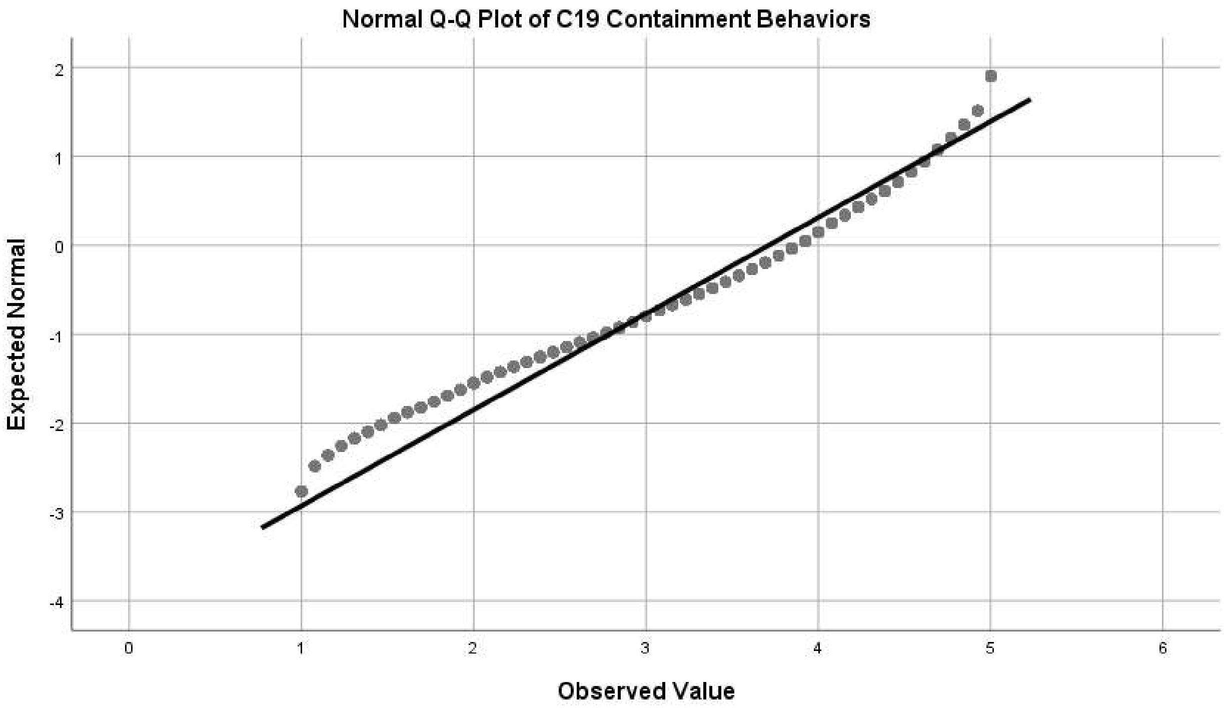

Notes. Q-Q plot displays the observed and expected (normal) values for self-reported support for COVID-19 containment behaviors across $N = 12{,}758$ independent respondents.

Data and Code Availability Statement

The codes for running the alternative models and the data underlying all analyses presented in the Supplementary Information are available at https://osf.io/kws9x/files[2].

Supplementary References

1. Oshchepkov, A. & Shirokanova, A. Bridging the gap between multilevel modeling and economic methods. *Social Science Research,* ***104*****,** 102689 (2022).

2. Karakulak, A. Contrasting the Effects of Pro-social Motivation versus Fear of Covid-19 as a Function of Governmental Trust on Practicing Covid-19 Containment Behaviors Across Countries. OSF Files at https://osf.io/kws9x/files/ (2023).
