# Supplementary material for: Trust in government moderates the association between fear of COVID-19 as well as empathic concern and preventive behaviour
Source: Commun Psychol. 2023 Dec 15;1:43. doi: 10.1038/s44271-023-00046-5 (PMC11332001; doi:10.1038/s44271-023-00046-5)
Supplement: Supplementary file 3 — Reporting Summary [file 44271_2023_46_MOESM3_ESM.pdf]

nature portfolio

Corresponding author(s): Arzu Karakulak

Last updated by author(s): Nov 9, 2023

Reporting Summary

Nature Portfolio wishes to improve the reproducibility of the work that we publish. This form provides structure for consistency and transparency in reporting. For further information on Nature Portfolio policies, see our Editorial Policies and the Editorial Policy Checklist.

Statistics

For all statistical analyses, confirm that the following items are present in the figure legend, table legend, main text, or Methods section.

n/a	Confirmed	
☐	☒	The exact sample size (*n*) for each experimental group/condition, given as a discrete number and unit of measurement
☐	☒	A statement on whether measurements were taken from distinct samples or whether the same sample was measured repeatedly
☒	☐	The statistical test(s) used AND whether they are one- or two-sided<br>*Only common tests should be described solely by name; describe more complex techniques in the Methods section.*
☐	☒	A description of all covariates tested
☐	☒	A description of any assumptions or corrections, such as tests of normality and adjustment for multiple comparisons
☐	☒	A full description of the statistical parameters including central tendency (e.g. means) or other basic estimates (e.g. regression coefficient) AND variation (e.g. standard deviation) or associated estimates of uncertainty (e.g. confidence intervals)
☒	☐	For null hypothesis testing, the test statistic (e.g. *F*, *t*, *r*) with confidence intervals, effect sizes, degrees of freedom and *P* value noted<br>*Give P values as exact values whenever suitable.*
☒	☐	For Bayesian analysis, information on the choice of priors and Markov chain Monte Carlo settings
☐	☒	For hierarchical and complex designs, identification of the appropriate level for tests and full reporting of outcomes
☐	☒	Estimates of effect sizes (e.g. Cohen's *d*, Pearson's *r*), indicating how they were calculated

Our web collection on statistics for biologists contains articles on many of the points above.

Software and code

Policy information about availability of computer code

Data collection	Data collection at the different study locations was realized via self-report online surveys that were created with survey tools such as Google Forms or Qualtrics. The obtained data was then combined into an overall dataset that has been made available as an Excel file at https://osf.io/kws9x/files/.
Data analysis	We analyzed the data obtained from this study with the open source software jamovi and provided the analysis outputs inclduing the codes to reproduce the analyses at https://osf.io/kws9x/files/

For manuscripts utilizing custom algorithms or software that are central to the research but not yet described in published literature, software must be made available to editors and reviewers. We strongly encourage code deposition in a community repository (e.g. GitHub). See the Nature Portfolio guidelines for submitting code & software for further information.

Data

Policy information about availability of data

All manuscripts must include a data availability statement. This statement should provide the following information, where applicable:

- Accession codes, unique identifiers, or web links for publicly available datasets
- A description of any restrictions on data availability
- For clinical datasets or third party data, please ensure that the statement adheres to our policy

The data generated and/or analyzed during the current study are available on the Open Science Framework repository, https://osf.io/kws9x/. The World Values

Survey data are available at https:// www.worldvaluessurvey.org/wvs.jsp. Scores on HDI were extracted from https://hdr.undp.org/data-center/documentation-and-downloads. Data about hospital beds per thousand were extracted from https://ourworldindata.org/grapher/hospital-beds-per-1000-people. Data about government stringency level were extracted from https://covidtracker.bsg.ox.ac.uk/stringency-map. The scores for daily new COVID-19 cases and deaths represent a 7-days rolling average per million people and were extracted from https://ourworldindata.org/explorers/coronavirus-data-explorer?uniformYAxis=0&pickerSort=asc&pickerMetric=location&Metric=Cases+and+deaths&Interval=7-day+rolling+average&Relative+to+Population=true&Color+by+test+positivity=false.

Human research participants

Policy information about studies involving human research participants and Sex and Gender in Research.

Reporting on sex and gender	Throughout the manuscript we only asked respondents to self-identify their gender that is shaped by social and cultural context. Answer options included man, woman, none of the above/other,and non-binary. At no stage, the respondents were asked to indicate their biological sex. Hence, we only used the expression of gender in its appropriate way.
Population characteristics	The data were collected from adult participants aged above 18 years in 34 different countries. No other inclusion criteria were defined. The final dataset consisted of N= 12,758 individual responses (Mage = 26.9; 67% female) from adult participants, collected over a period of approximately one year (from February 2021 to December 2021). Table 2 in the MS shows the distribution across study location. For descriptive reasons, respondents were further asked to report their nationality and their ethnicity, in the format of an open-ended question. This question was not mandatory; and no exclusions or additional analyses were made with respect to respondents' nationality or ethnicity.
Recruitment	The data collection was conducted within the framework of a large-scale collaborative project investigating "International and Multidimensional Perspectives on the Impact of COVID-19 across Generations (IMPACT-C19)", spanning across different nations around the globe. The project focused specifically on the impact, perceptions, and experiences of COVID-19 among young people and established adults in an international perspective (Rivera, 2021). Participating researchers were invited to: a) obtain ethics approval from their home institution and b) collect data using the convenience sampling method. Informed consent prior to voluntary participation was obtained from each participant. Data from countries that provided at least 150 complete responses (by January 2022) were included into the analyses.
Ethics oversight	The current study protocol has been reviewed and approved by the Institutional Review Board of Bahcesehir University (IRB protocol number: E-8755). When not declared as exempt, approvals have additionally been obtained from the local institutional review boards of all other involved countries. Written informed consent was obtained from each participant prior to completing the research. Only participants that agreed to the study's conditions were allowed to proceed with the research. Participation to this research was voluntary and no monetary compensation was given to respondents for completing this research.

Note that full information on the approval of the study protocol must also be provided in the manuscript.

Field-specific reporting

Please select the one below that is the best fit for your research. If you are not sure, read the appropriate sections before making your selection.

☐ Life sciences ☒ Behavioural & social sciences ☐ Ecological, evolutionary & environmental sciences

For a reference copy of the document with all sections, see nature.com/documents/nr-reporting-summary-flat.pdf

Behavioural & social sciences study design

All studies must disclose on these points even when the disclosure is negative.

Study description	This is a cross-sectional quantitative multi-national study using the convenience sampling method to assess adult respondents' views, perceptions, feelings, beliefs and behaviors in times of the COVID-19 pandemic. The data collection has been realized via online self-report surveys.
Research sample	The sample consisted of adult respondents > 18 years with access to internet and the ability to complete an online survey (all respondents are thus literate). No other inclusion criteria were defined.
Sampling strategy	The convenience sampling procedure was used at each study location.
Data collection	Data collection was realized via online self-report surveys using survey platforms such as Google Forms or Qualtrics.<br>The entire survey administered to the participants consisted of socio-demographic background questions, and both already established and newly developed scales to assess concepts associated with COVID-19 and its impacts. Examples of the concepts are COVID-19 threat perception, fear of COVID-19, support for COVID-19 containment behaviors, national identity, hope, mindfulness, empathic concern, and religiousness. The survey was first developed in English and then translated and adapted to the local contexts (where necessary) by using the committee approach (Beaton et al., 2000; Brislin, 1980). The survey was anonymous, and completed on a voluntary basis (within 25 minutes approximately). The entire survey containing all scales is available at https://osf.io/kws9x/.

Timing	Data collection took place between February 2021 and December 2021
Data exclusions	Answers of respondents younger than 18 years, or responses with incomplete answers and missing values on any of the study's variables were deleted listwise from the dataset. Only countries that provided at least 150 complete answers by January 2022 were included into the analyses.
Non-participation	The raw dataset without any data exclusions by January 20th 2022 comprised data of 27787 responses. As oulined above, we first deleted responses from all respondents that were aged below 18 (or did not indicate any age; N = 4951); and then excluded all data listwise with any missing values on the study variables and their items (N = 8161). Finally, data from 22 countries with less than 150 complete responses were removed (N = 1917) resulting in the final dataset based on which the analyses were performed. These countries were Czech Republic, Slovenia, Taiwan, USA, Costa Rica, Niger, Zambia, Zimbabwe, Afghanistan, Dominican Republic, Uganda, Mozambique, Argentina, Kazakhstan, Kosovo, Albania, North Macedonia, Armenia, Guatemala, Bosnia Herzegovina, Qatar and Chile.
Randomization	not applicable

Reporting for specific materials, systems and methods

We require information from authors about some types of materials, experimental systems and methods used in many studies. Here, indicate whether each material, system or method listed is relevant to your study. If you are not sure if a list item applies to your research, read the appropriate section before selecting a response.

Materials & experimental systems

n/a	Involved in the study	
☒	☐	Antibodies
☒	☐	Eukaryotic cell lines
☒	☐	Palaeontology and archaeology
☒	☐	Animals and other organisms
☒	☐	Clinical data
☒	☐	Dual use research of concern

Methods

n/a	Involved in the study	
☒	☐	ChIP-seq
☒	☐	Flow cytometry
☒	☐	MRI-based neuroimaging
